# Cecal metagenome and mucosal transcriptome of broilers after an enteric challenge and fed diets with different fiber types and concentrations^1^

**DOI:** 10.1016/j.psj.2026.107151

**Published:** 2026-05-22

**Authors:** R.W. Tabish, Y. Lin, S.J. Rochell, W.J. Pacheco, M.A. Bailey, W.A. Dozier, K. Robinson, R. Hauck

**Affiliations:** a Department of Poultry Science, Auburn University, Auburn, AL 36849, USA; b USDA-ARS Poultry Research Unit, Mississippi State, MS, 39762, USA; c Department of Pathobiology, Auburn University, Auburn, AL 36849, USA

**Keywords:** Dietary fiber, enteric infection, cecal microbiome, host transcriptomics, multi‑omics integration

## Abstract

This study evaluated the effects of dietary fiber supplementation on broiler gut health during a subclinical enteric challenge. Birds were assigned to either an unchallenged control or a challenged control, followed by six dietary treatments applied to challenged birds. These treatments included 3% oat hulls (OH), 3% soy hulls (SH), and four combinations of 1.5% OH or SH with 1.5% wheat middlings (WM) or sugar beet pulp (SBP). A randomized complete block design was used with 2,160 day-old YP × Ross 708 male broiler chicks allocated to eight treatments, each with nine replicate floor pens and 30 birds per pen. Birds were inoculated with *Eimeria* followed by *Clostridium perfringens*, and cecal samples were collected at 21 days of age for shotgun metagenomic and transcriptomic analyses. The enteric challenge significantly reduced microbial diversity, depleted butyrate-producing bacteria, and enriched pathways associated with bacterial growth and virulence while triggering inflammatory signaling and suppressing proliferative pathways in the host. Supplementation with dietary fiber modulated these responses through distinct yet complementary mechanisms. The group receiving OH with WM enriched butyrate-producing bacteria, including *Faecalibacterium prausnitzii*, reduced *C. perfringens* abundance, and downregulated inflammatory pathways. Birds fed OH with SBP showed increased populations of lactic acid producing bacteria and *Bifidobacterium animalis* while suppressing TNFα, NF-κB and IFNγ signaling. Diets containing SH combinations enhanced metabolic pathways related to pyruvate fermentation and stachyose degradation, primarily driven by *Lactobacillus* species. Despite having distinct microbial compositions, all fiber treatments restored epithelial proliferation pathways in the host transcriptome, indicating convergent potentially beneficial effects on intestinal health. Integration of bacteriome and transcriptome data revealed coordinated relationships between specific bacterial species, including *Stutzerimonas stutzeri, Bacteroides caecae*, and *Eubacteriaceae bacterium* ES3, and host genes involved in immune function and energy metabolism. These findings provide a mechanistic framework for developing targeted nutritional strategies using specific fiber combinations to enhance gut resilience in antibiotic-free broiler production systems.

## Introduction

Subclinical necrotic enteritis (**NE**) caused by the co-infection of *Eimeria maxima* and *Clostridium perfringens* is one of the most economically significant enteric diseases in the global poultry industry, with an economic burden that has increased substantially over the years. The first global estimate placed the annual losses at approximately US$2 billion (Van der Sluis, 2000). A subsequent reassessment based on producer surveys and field data increased this estimate to approximately US$6 billion annually by 2015 (Wade and Keyburn, 2015), which is consistent with the economic analysis of subclinical NE impacts in broiler chickens documented by Skinner et al. (2010). According to FAOSTAT data, the total number of chickens slaughtered globally increased from approximately 64 billion in 2015 to 76 billion in 2023 (FAOSTAT, 2025), representing an 18.8% increase. When combined with cumulative inflation of approximately 36-37% from 2015 to 2025 (CPI inflation calculator, 2025) the current global economic burden of NE is estimated at approximately US$9.8-10.2 billion annually, equivalent to roughly US$0.13 per bird slaughtered. This increased economic burden of NE over years necessitates effective non-antibiotic control measures for antibiotic-free poultry production systems.

Dietary fiber (**DF**) offers a promising nutritional strategy. Insoluble fibers, such as 2-3% oat hulls, stimulate gizzard development, lower luminal pH, and improve gut motility, which reduces the risk of pathogen colonization in the distal gut (González-Alvarado et al., 2010; Mateos et al., 2012). Mixed solubility fibers, as 7% inclusion of soy hulls, are fermented into beneficial short-chain fatty acids (**SCFAs**) that enrich populations of *Lactobacillus* and *Bifidobacterium*, and can increase digesta viscosity, physically impeding pathogen access to the intestinal mucosa (Min, 2013; Yang et al., 2020a). Wheat middlings, another fibrous ingredient comprising non-starch polysaccharides (**NSPs**) and mixed dietary fiber (5-15% inclusion), similarly stimulate gizzard development and increase cecal SCFA production through bacterial fermentation. Moderate wheat middlings inclusion enhances intestinal barrier function and villus height without negatively impacting growth performance, particularly when supplemented with NSP-degrading enzymes such as xylanase (Njeri et al., 2023; Wang et al., 2024). Conversely, sugar beet pulp (3-7% inclusion), a fiber source rich in soluble pectin, must be used cautiously in antibiotic-free diets. While it provides a fermentable substrate, its high soluble NSP content significantly increases digesta viscosity and slows the feed passage rate, which impairs nutrient absorption and reduces villus height (Jiménez-Moreno et al., 2013; Sadeghi et al., 2015).

A synergistic approach using combined fiber types is supported by findings where supplementing pullet diets with 1% mixed soluble and insoluble DF increased the relative weight of the bursa of Fabricius by 14% and the area of Peyer’s patches by 50%, while also enhancing T and B-lymphocyte proliferation, demonstrating a broad improvement in gut associate lymphoid tissue (**GALT**) development and function (Hussein et al., 2017).

Microbial metabolites such as SCFAs are important for gut health, such as butyrate which serves as a primary energy source for colonocytes and enhancing intestinal barrier integrity by inducing the IL-10 receptor α subunit, which in turn represses the permeability-promoting tight-junction protein claudin-2 (Zheng et al., 2017). Furthermore, DF can directly modulate host immune responses; for instance, dietary pectin has been shown to temper the inflammatory cascade during coccidial infection by altering the expression of key cytokines such as IL-12 in the ileal mucosa and IFNγ in the cecal tonsils (Wils-Plotz et al., 2013). However, a systematic evaluation of how specific insoluble-soluble DF combinations modulate gut microbiome, its metabolism and the host transcriptomic response simultaneously during a complex *Eimeria-C. perfringens* co-infection is currently lacking.

This study was designed to address this gap by investigating six dietary fiber treatments administered to challenged birds: (1) 3% oat hulls (OH), representing a predominantly insoluble fiber source; (2) 3% soy hulls (SH), containing both insoluble and fermentable fiber fractions; and (3-6) four combinations consisting of 1.5% insoluble and 1.5% more fermentable fiber sources using wheat middlings (WM) or sugar beet pulp (SBP), alongside challenged and unchallenged control groups without supplemental fiber. We hypothesized that specific insoluble-soluble DF combinations would restore the microbial homeostasis by providing substrates for butyrate producers, competitively excluding pathogens, and modulating the host immune response. By integrating shotgun metagenomics and host transcriptomics, this study aims to mechanistically link fiber composition to gut health modulation in antibiotic-free systems.

## Materials and methods

### Experimental design, animals, and housing

The experimental protocol was approved by the Auburn University Institutional Animal Care and Use Committee (2023-5223). The study employed a randomized complete block design with pen location as the blocking factor. A total of 2,160-day-old YP × Ross 708 male broiler chicks, obtained from a commercial hatchery, were randomly allocated to 8 dietary treatment groups. Each treatment consisted of 9 replicate floor pens containing 30 birds per pen. Birds were raised on wood shaving litter in floor pens (2.79 m²) equipped with hanging feeders and nipple drinker line. Feed and water were provided *ad libitum* throughout the 35-d experimental period. The initial ambient temperature was maintained at 33°C at placement and gradually decreased to 20°C by d 32. Lighting schedule consisted of 23L:1D for the first 7 d, followed by 20L:4D for the remainder of the experiment. Light intensity was managed at 30, 10, and 5 lux during d 1 to 7, d 8 to 14, and d 15 to 35, respectively.

### Experimental diets

Corn-soybean meal-based diets were formulated to meet Aviagen broiler nutrient requirements (Aviagen, 2022), with adequate levels of amino acids, vitamins, and minerals. Eight dietary treatments were evaluated: an unchallenged control (NC) and a challenged control (PC), both without supplemental fiber, plus six challenged groups receiving diets supplemented with different fiber sources. The fiber sources included oat hulls (OH; 82.07% NDF (Neutral Detergent Fiber), 46.57% ADF (Acid Detergent Fiber), and 35.49% hemicellulose), sugar beet pulp (SBP; 35.69% NDF, 22.88% ADF, and 12.80% hemicellulose), soy hulls (SH; 63.79% NDF, 46.67% ADF, and 17.12% hemicellulose), and wheat middlings (WM; 38.52% NDF, 12.20% ADF, and 26.32% hemicellulose). Challenged groups received diets containing either 3% OH, 3% SH, or combinations of 1.5% of each fiber source, including oat hulls with wheat middlings (OH-WM), oat hulls with sugar beet pulp (OH-SBP), soy hulls with wheat middlings (SH-WM), or soy hulls with sugar beet pulp (SH-SBP). Experimental diets were manufactured at the Auburn University Feed Mill at the Charles Miller Research and Education Center. The fiber sources were obtained from a supplier and distributor of feed ingredients and agricultural byproducts (Cerco Group) and were selected because they represent commonly used feed commodities that are readily available for practical application in commercial poultry feed formulations.

### Enteric challenge model

Birds in the challenged treatment groups were orally gavaged on d 14 with 1 mL of a commercial trivalent coccidial vaccine (Advent®, Huvepharma, Sofia, Bulgaria) administered at 10 times the manufacturer’s recommended dose. This provided approximately 2,300 oocysts of *E. maxima* and a combined 9,600 oocysts of *E. acervulina* and *E. tenella* per bird. Birds in the unchallenged control group received 1 mL of sterile phosphate-buffered saline. The dietary transition from starter to grower feed occurred on d 17. On d 18, birds in the challenged groups were gavaged with 1 mL of brain heart infusion (**BHI**) broth culture containing a NetB-negative strain of *C. perfringens* (1 × 10⁸ CFU/mL). Birds in the unchallenged control group received a sham gavage of 1 mL of sterile BHI broth (Becton, Dickinson and Company, Sparks, MD).

### Sample collection

At d 21, i.e. 3 d post *C. perfringens* challenge, euthanasia of 8 birds per pen was performed using CO₂ asphyxiation with subsequent cervical dislocation following American Veterinary Medical Association guidelines (AVMA, 2020). Cecal contents weighing approximately 2 g were collected from 1 bird per pen for metagenomic analysis, placed immediately on ice, then transferred to −80°C storage. From the same bird, mucosal scrapings were obtained from cecal segments measuring 5-10 cm in length for transcriptomic evaluation. These mucosal samples were preserved in RNAlater (Thermo Fisher Scientific™, Waltham, MA), maintained at 4°C for a 24-h period, after which the preservation solution was removed, and samples were stored at −80°C until further processing.

### Metagenomic analysis

Genomic DNA was extracted from approximately 200 mg of cecal contents using the QIAamp DNA Stool Kit (Qiagen, Hilden, Germany) according to the manufacturer’s instructions. DNA concentration and purity were verified using an Implen NanoPhotometer® N60 (Implen GmbH, Munich, Germany). Shotgun metagenomic sequencing was outsourced to Azenta Life Sciences (South Plainfield, NJ). Sequencing libraries were prepared and run on an Illumina platform using a 2 × 150 bp paired-end configuration, targeting ∼10 Gb per sample, with ≥ 80% of bases exceeding a Phred quality score of Q30. Raw sequencing data are available in the NCBI Sequence Read Archive (BioProject PRJNA1187240).

Raw reads were assessed with FastQC (Andrews, 2010), and low-quality bases and adapters were trimmed using Trimmomatic v0.39 (Bolger et al., 2014). Host-derived sequences were removed by aligning reads to the *Gallus gallus* reference genome (GRCg7b) using HISAT2 v2.2.0 (Kim et al., 2019b). Non-host reads were retained for downstream analyses. Taxonomic classification was performed with Kraken2 (Wood et al., 2019) against the comprehensive BLAST core nucleotide (core_nt) database. For a read to be assigned a taxonomic label, a stringent criterion was applied requiring at least 20% of its constituent k-mers to map to a single taxon (confidence threshold of 0.2), thereby minimizing ambiguous or low-confidence assignments. Following initial classification, Bracken (Lu et al., 2017) was employed to refine these assignments and generate more accurate species-level abundance profiles. This was accomplished by using a Bayesian statistical model specifically parameterized for our 150 bp read length to re-distribute reads from higher taxonomic ranks to the most probable species. Functional annotation and pathway profiling were performed using HUMAnN v3.6 (Beghini et al., 2021) with MetaPhlAn for integrated taxonomic profiling.

### Transcriptome analysis

Total RNA was isolated from approximately 15 mg of cecal mucosal tissue using the RNeasy Mini Kit (Qiagen, Hilden, Germany) incorporating bead-mill homogenization and on-column DNase digestion. RNA purity was evaluated using an Implen NanoPhotometer® N60 (Implen GmbH, Munich, Germany), with samples having A260/280 ≥ 1.8 considered acceptable. RNA integrity was verified using an Agilent 5400 Fragment Analyzer (Agilent Technologies, Ramsey, MN), and samples with RNA integrity number ≥ 4.0 were preferred for library preparation. Libraries were prepared with poly-A enrichment, and RNA sequencing was outsourced to Novogene Corporation Inc. (Sacramento, CA), performed on an Illumina NovaSeq X Plus platform to generate ∼30 million 150 bp paired-end reads per sample. Sequencing data are available at NCBI under BioProject PRJNA1187003.

Bioinformatic processing involved quality assessment with FastQC and adapter trimming using Trimmomatic v0.39. Clean reads were aligned to the *Gallus gallus* reference genome (GRCg7b) using HISAT2, and gene-level quantification was performed using HTSeq-count v1.13.3 (Anders and Pyl, 2015). Differential expression analysis was conducted in R v4.5.0 (R Core Team, 2023) using DESeq2 (Love and Huber, 2014). Pairwise comparisons were conducted to evaluate both the effects of the enteric challenge and the impact of dietary fiber supplementation. The NC group was used as the reference to assess the effect of the challenge (PC vs. NC). Subsequently, the PC group served as the reference for evaluating the effects of dietary fiber treatments, with each fiber-supplemented group compared against the PC. Only genes with cumulative counts ≥ 20 across relevant samples were retained. Differentially expressed genes (**DEGs**) were defined as those with adjusted *P* < 0.05 and |log₂ fold change| > 1. For pathway enrichment, genes were preranked by the metric sign (log_2_ (FoldChange)) × (-log_10_ (*P*-value)) and analyzed using the Preranked **GSEA** module (Gene Set Enrichment Analysis software, Broad Institute). Hallmark pathways with false discovery rate (**FDR**) q-values ≤ 0.05 were considered significantly enriched and are reported with their normalized enrichment scores (**NES**).

### Bacteriome and host gene correlation analysis

To investigate bacteriome-host gene expression correlation, common samples from both datasets were identified and analyzed in R. Species-level abundances were normalized using a Centered Log-Ratio (**CLR**) transformation and host gene counts were normalized using the variance stabilizing transformation (**VST**). Overall concordance between the normalized datasets was assessed using Procrustes analysis. To identify correlated sets of microbes and genes, a Sparse Canonical Correlation Analysis (**sCCA**) was conducted on standardized data. To identify specific microbial taxa associated with individual host genes, a gene-by-gene Lasso regression was performed modeling each gene’s VST-normalized expression as a response to CLR-transformed microbial abundances. Significant associations were then filtered and visualized as a host-microbe interaction network.

### Statistical analysis

All statistical analyses were performed in R (v4.5.0). The experiment followed a randomized complete block design, with nine replicate pens per treatment. The individual bird (one per pen) represented the experimental unit for metagenomic and transcriptomic analyses. Microbial alpha-diversity data was evaluated using the Kruskal-Wallis test, followed by Dunn’s post-hoc test with Bonferroni correction when significant differences (*P* ≤ 0.05) were detected. Preliminary testing confirmed no significant block effects, allowing for a simplified model. Microbial beta-diversity was analyzed by **PERMANOVA** (permutational multivariate analysis of variance) using the adonis function in the vegan package (Oksanen et al., 2025). Differential abundance of taxa was determined using DESeq2, with the PC group as the reference. Taxa were considered significantly differentially abundant at adjusted *P* < 0.05 (Benjamini-Hochberg correction), and results are presented with log₂ fold changes. For microbial functional pathway analysis, HUMAnN3-derived pathway abundance tables were filtered to remove unmapped, unintegrated, and stratified pathways prior to statistical testing. Pairwise comparisons against the PC group were performed using DESeq2, with significance defined at *P* < 0.05. Statistical approaches specific to host transcriptomic data are described above.

## Results

### Cecal bacteriome

**Fig. 1:**
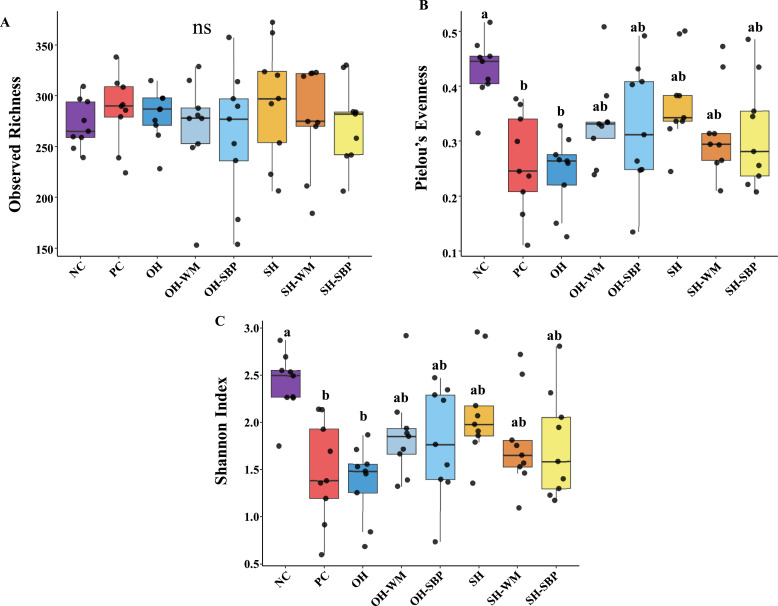
Effect of different dietary fiber sources on cecal bacteriome alpha diversity. Box plots represent three alpha diversity metrics: (A) observed features (richness), (B) Pielou’s evenness, and (C) the Shannon diversity index. In each plot, the horizontal line indicates the median, box boundaries represent the interquartile range, and whiskers extend to 1.5 times the interquartile range. Individual data points are overlaid. For panels B and C, superscripts (a, b) denote significant differences between treatment groups; box plots not sharing a letter are significantly different (*P* < 0.05) as determined by the Kruskal-Wallis test followed by Dunn’s post hoc test. Treatment groups are as follows: NC, Negative Control (unchallenged); PC, Positive Control (challenged); and challenged groups receiving diets containing OH, Oat Hulls; SH, Soy Hulls; OH-WM, Oat Hulls with Wheat Middling; OH-SBP, Oat Hulls with Sugar Beet Pulp; SH-WM, Soy Hulls with Wheat Middling; and SH-SBP, Soy Hulls with Sugar Beet Pulp.

**Fig. 2:**
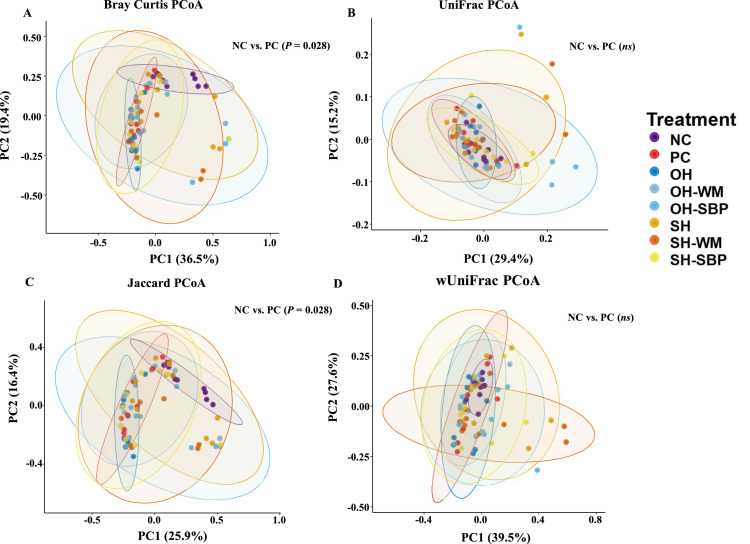
Beta diversity of cecal bacterial communities in response to dietary fiber supplementation. Principal Coordinate Analysis (PCoA) plots were generated based on four distance metrics: (A) Bray-Curtis dissimilarity, (B) unweighted UniFrac distance, (C) Jaccard dissimilarity, and (D) weighted UniFrac distance. Each point represents the microbial community of an individual sample, colored by its respective treatment group. Ellipses represent the 95% confidence interval for the centroid of each group. The percentage of variation explained by each principal coordinate axis is indicated in brackets. P-values shown on each plot are derived from pairwise PERMANOVA tests comparing the Negative Control (NC) and Positive Control (PC) groups (ns, not significant, *P* > 0.05). Treatment groups are as follows: NC, Negative Control (unchallenged); PC, Positive Control (challenged); and challenged groups receiving diets containing OH, Oat Hulls; SH, Soy Hulls; OH-WM, Oat Hulls with Wheat Middling; OH-SBP, Oat Hulls with Sugar Beet Pulp; SH-WM, Soy Hulls with Wheat Middling; and SH-SBP, Soy Hulls with Sugar Beet Pulp.

**Fig. 3:**
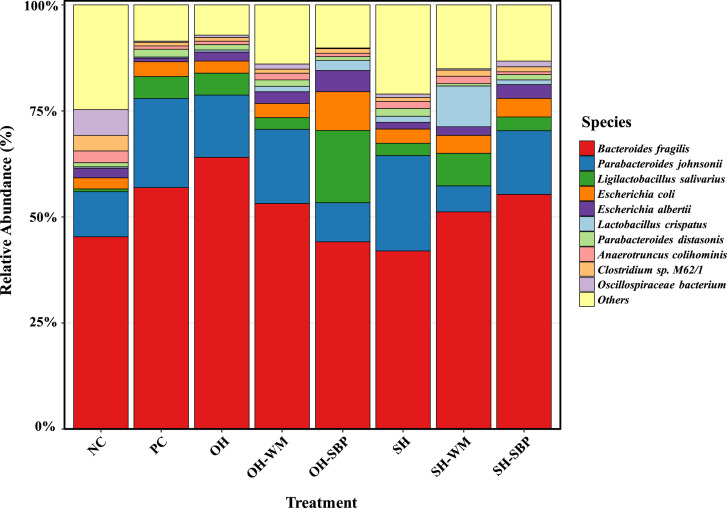
Relative abundance of the top 10 bacterial species in cecal samples across all treatment groups. This stacked bar plot illustrates the mean relative abundance of the most prevalent bacteria at the species level. Each bar represents a distinct treatment group, and the colored segments correspond to the relative abundance of the top 10 identified species. The ‘Others’ category comprises all remaining, less abundant species not ranked in the top 10. Treatment groups are as follows: NC, Negative Control (unchallenged); PC, Positive Control (challenged); and challenged groups receiving diets containing OH, Oat Hulls; SH, Soy Hulls; OH-WM, Oat Hulls with Wheat Middling; OH-SBP, Oat Hulls with Sugar Beet Pulp; SH-WM, Soy Hulls with Wheat Middling; and SH-SBP, Soy Hulls with Sugar Beet Pulp.

The shotgun metagenomic sequencing of cecal samples yielded an average of approximately 36 million reads per sample, ensuring sufficient depth for comprehensive microbial profiling and diversity analysis (Figure S1). The enteric challenge significantly altered the composition of the cecal microbiome. Alpha diversity analysis revealed that the challenge reduced microbial community evenness (Pielou’s evenness, *P* = 0.010) and diversity (Shannon index, *P* = 0.016) compared to the unchallenged control (Fig. 1). Beta diversity analysis also showed a significant shift in the overall microbial community structure between the NC and PC groups, as measured by both Bray-Curtis (*P* = 0.028) and Jaccard (*P* = 0.028) distances (Fig. 2). Fiber supplementation did not result in significant changes to either alpha or beta diversity compared to the PC group (*P* > 0.05). The relative abundance of the most dominant bacterial species was altered by the enteric challenge (Fig. 3). In the unchallenged group, *Bacteroides fragilis* was the predominant species (45.3%), followed by *Parabacteroides johnsonii* (10.6%). The enteric challenge shifted the bacterial profile, increasing the relative abundance of *B. fragilis* (56.9%) and *P. johnsonii* (21.0%).

**Fig. 4:**
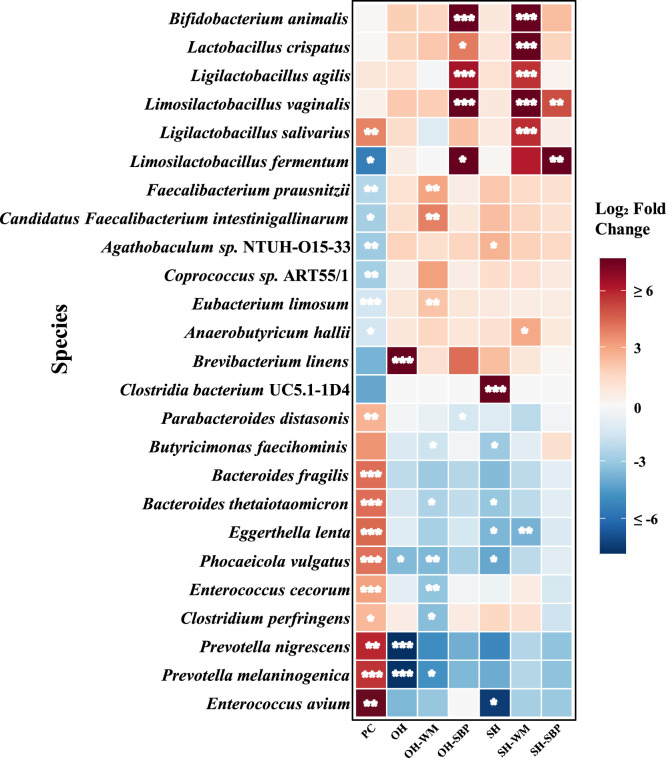
Top 25 differentially abundant bacterial taxa in cecal contents across dietary treatment groups. The heatmap displays the Log₂ fold change of species identified as significantly different in at least one comparison (*P*_*adj*_ < 0.05). Rows correspond to individual bacterial species, and columns represent pairwise comparisons of each treatment group. Colors indicate the magnitude of fold change relative to the PC, with red shades denoting higher relative abundance and blue shades denoting lower relative abundance. For the first comparison (PC vs. NC), the NC serves as the reference, and the direction of regulation reflects changes in the PC. Asterisks within the cells represent the level of statistical significance for that specific comparison: *P_*adj*_ < 0.05, **P_*adj*_ < 0.01, and ***P_*adj*_ < 0.001. Treatment groups are as follows: PC, Positive Control (challenged); and challenged groups receiving diets containing OH, Oat Hulls; SH, Soy Hulls; OH-WM, Oat Hulls with Wheat Middling; OH-SBP, Oat Hulls with Sugar Beet Pulp; SH-WM, Soy Hulls with Wheat Middling; and SH-SBP, Soy Hulls with Sugar Beet Pulp.

Based on differential abundance analysis, the enteric challenge significantly (*P_adj_* < 0.001, unless indicated otherwise) decreased the differential abundance of several butyrate-producing bacteria, including *Faecalibacterium prausnitzii* (log₂FC = −1.78, *P_adj_* = 0.006), *Anaerobutyricum hallii* (log₂FC = −1.15, *P_adj_* = 0.036), and *Coprococcus* sp. ART55/1 (log₂FC = −1.72) compared to the unchallenged control (Fig. 4). Conversely, the challenge enriched numerous species within the Bacteroidales order, including *Bacteroides fragilis* (log₂FC = 2.69), *B. thetaiotaomicron* (log₂FC = 3.34), *Phocaeicola vulgatus* (log₂FC = 3.29), and *Parabacteroides distasonis* (log₂FC = 2.27). The supplementation of OH in the diet of the challenged group increased the abundance of *Brevibacterium linens* (log₂FC = 22.87) and decreased several species, including *Phocaeicola vulgatus* (log₂FC = −2.65, *P_adj_* = 0.010), *Prevotella melaninogenica* (log₂FC = −5.94), and *Prevotella nigrescens* (log₂FC = −6.38). The combination of OH with WM increased the abundance of butyrate-producers, including *Faecalibacterium prausnitzii* (log₂FC = 2.39, *P_adj_* = 0.007), *Candidatus Faecalibacterium intestinigallinarum* (log₂FC = 3.06, *P_adj_* = 0.007), and *Eubacterium limosum* (log₂FC = 3.48, *P_adj_* = 0.011), while decreasing *C. perfringens* (log₂FC = −2.55, *P_adj_* = 0.015), *Enterococcus cecorum* (log₂FC = −2.39, *P_adj_* = 0.007), and *Butyricimonas faecihominis* (log₂FC = −2.20, *P_adj_* = 0.007) compared to PC. The OH, along with SBP supplementation, increased the differential abundance of *Bifidobacterium animalis* (log₂FC = 23.69), *Ligilactobacillus agilis* (log₂FC = 4.99), *Limosilactobacillus vaginalis* (log₂FC = 6.14), and *Lactobacillus crispatus* (log₂FC = 3.15, *P_adj_* = 0.019). Supplementation of SH alone significantly increased *Clostridia bacterium UC5.1-1D4* (log₂FC = 21.92), and *Agathobaculum sp.* (log₂FC = 2.07, *P_adj_* = 0.049), while decreasing *B. thetaiotaomicron* (log₂FC = −2.32, *P_adj_* = 0.049), *Phocaeicola vulgatus* (log₂FC = −3.10, *P_adj_* = 0.024), *Butyricimonas faecihominis* (log₂FC = −2.62, *P_adj_* = 0.024), and *Enterococcus avium* (log₂FC = −5.71, *P_adj_* = 0.024). The treatment receiving SH, along with WM, induced enrichment of various lactic acid bacteria, including *Lactobacillus crispatus* (log₂FC = 8.20), *Ligilactobacillus agilis* (log₂FC = 4.44), and *Limosilactobacillus vaginalis* (log₂FC = 8.04), while also increasing the butyrate-producer *Anaerobutyricum hallii* (log₂FC = 2.30, *P* = 0.044) and decreasing *Eggerthella lenta* (log₂FC = −2.85). Finally, the combination of SH with SBP in the diet of the challenged group increased *Limosilactobacillus fermentum* (log₂FC = 7.84, *P_adj_* = 0.009), and *Limosilactobacillus vaginalis* (log₂FC = 3.96, *P_adj_* = 0.009) compared to PC.

### Cecal mycobiome

**Fig. 5:**
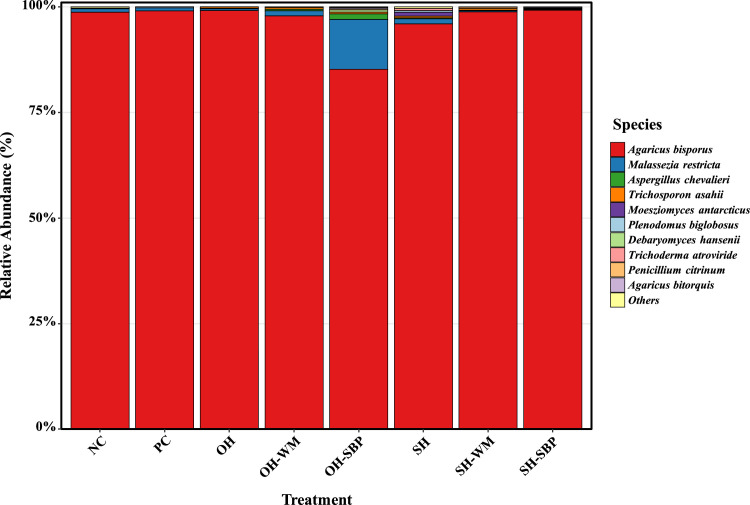
Relative abundance of the top 10 fungal species in cecal samples across all treatment groups. This stacked bar plot illustrates the mean relative abundance of the most prevalent fungi at the species level. Each bar represents a distinct treatment group, and the colored segments correspond to the relative abundance of the top 10 identified fungal species. The ‘Others’ category comprises all remaining, less abundant species not ranked in the top 10. Treatment groups are as follows: NC, Negative Control (unchallenged); PC, Positive Control (challenged); and challenged groups receiving diets containing OH, Oat Hulls; SH, Soy Hulls; OH-WM, Oat Hulls with Wheat Middling; OH-SBP, Oat Hulls with Sugar Beet Pulp; SH-WM, Soy Hulls with Wheat Middling; and SH-SBP, Soy Hulls with Sugar Beet Pulp.

Alpha and beta diversity analyses revealed no significant differences in cecal mycobiota composition among treatment groups (*P* > 0.05). The fungal community was predominantly composed of *Agaricus bisporus*, which accounted for more than 95% of the relative abundance across most groups (Fig. 5). The enteric challenge did not significantly alter the fungal profile, as both NC and PC groups maintained greater than 95% relative abundance of *A. bisporus*. However, specific fiber-supplemented diets numerically modulated the cecal mycobiota compared to the PC group. In the OH-SBP group, the relative abundance of *A. bisporus* decreased to 85.2%, accompanied by an increase in *Malassezia restricta* (11.8%) and *Aspergillus chevalieri* (1.3%). Similarly, the SH group exhibited an increased abundance of *M. restricta* (1.2%) and was uniquely characterized by the presence of *Moesziomyces antarcticus* (0.9%).

### Cecal virome

As the analysis was based on shotgun DNA sequencing, the characterization was limited to DNA viruses. At the taxonomic level, the cecal virome was overwhelmingly dominated by bacteriophages, with *Caudoviricetes sp.* constituting approximately 75-85% of the relative abundance across all treatment groups (Fig. S2). Other prevalent DNA virus taxa, albeit at much lower abundances, included various species of *Bacteriophage* and *Siphoviridae*. This general taxonomic profile, reflective of the bacteriophage community, remained remarkably consistent across all dietary treatments.

No significant differences were observed in the alpha or beta diversity of the cecal virome among treatment groups (*P* > 0.05). However, differential abundance analysis indicated that the enteric challenge significantly (*P_adj_* < 0.001) reduced the abundance of *Nonagvirus SE1Kor* (log₂FC = −21.29) compared to the unchallenged control. Fiber supplementation further modulated the virome composition slightly. The OH group increased the abundance of *Escherichia phage* ULINTec6 (log₂FC = 23.58), while the OH-WM group elevated the abundance of *Siphoviridae* sp. ctcj91 (log₂FC = 21.77) relative to PC. No other significant differences in viral taxa were detected among the remaining treatment groups following Benjamini-Hochberg correction. Notably, several other viral taxa showed differential abundance based on the unadjusted *P*-value, and these detailed results can be found in the supplementary material (Figure S3).

### Eimeria Abundance

Analysis of cecal *Eimeria* read counts revealed that the enteric challenge did not significantly increase the abundance of any *Eimeria* species in the PC group compared to the NC group (Table 1). However, a trend was observed for increased *Eimeria tenella* (*P* = 0.074) abundance in the PC group. When comparing fiber treatments to PC, none of the treatments significantly altered *Eimeria* counts for any of the three species (*E. acervulina, E. maxima* and *E. tenella*). Notably, when compared to the NC, several fiber-supplemented groups showed significantly higher counts of *E. acervulina* (OH-SBP, *P* = 0.008; OH-WM, *P* = 0.045; and SH, *P* = 0.005) and *E. tenella* (OH, *P* = 0.028 and SH, *P* = 0.045). In addition to these predominant species, DNA of other apicomplexans were detected in the cecum, including *Eimeria mitis, Eimeria necatrix*, and *Eimeria praecox*.

**Table 1:** Bracken-corrected raw read counts from Kraken2 taxonomic assignments across cecal samples of broilers provided diets with different dietary fiber sources and concentrations on d 21.

Apicomplexan species	Bracken-adjusted raw read counts based on Kraken2 classifications^1^							
	NC	PC	OH	OH-WM	OH-SBP	SH	SH-WM	SH-SBP
*Eimeria tenella*	44268	2829868	1215712	906970	543712	927096	297955	750838
*Eimeria maxima*	16254	12417	9716	10022	13561	8125	13398	13798
*Eimeria acervulina*	139	1137	1043	3162	4007	2918	1407	1528
*Eimeria praecox*	1905	1041	364	780	1042	670	2473	1867
*Eimeria necatrix*	15	748	327	197	91	385	62	159
*Eimeria mitis*	281	8	0	42	0	0	0	0
Uncultured *Eimeria*	0	3174	1014	951	0	536	0	494

1 Raw read counts represent the number of sequencing reads classified by Kraken2 at the species level using a confidence threshold of 0.2, requiring at least 20% of k-mers to support a taxonomic assignment, and subsequently corrected for abundance estimation using Bracken.

Very few archaeal reads were detected in the cecal samples, preventing meaningful comparative analysis of this domain.

### Microbial Functional Pathways

**Fig. 6:**
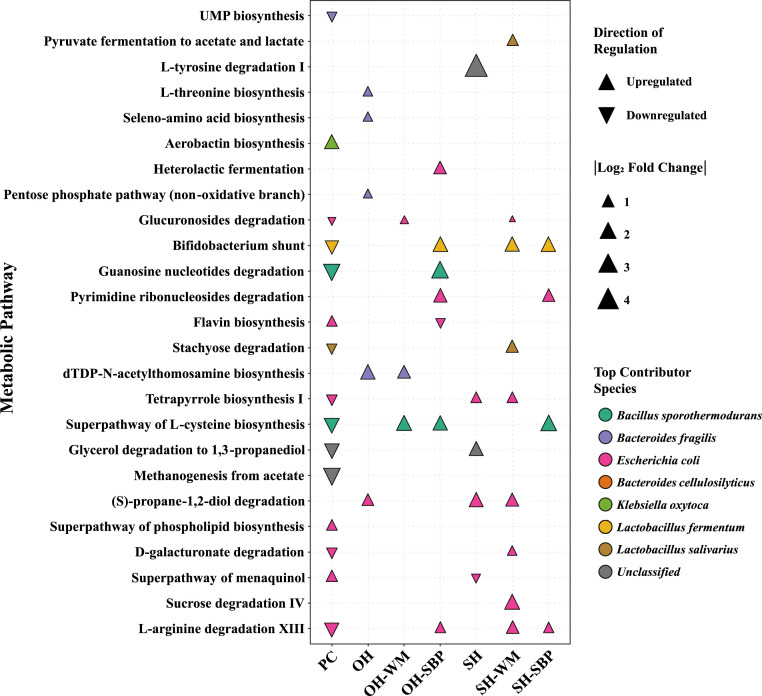
Differentially abundant metabolic pathways in cecal microbial communities. This plot displays 25 metabolic pathways identified using HUMAnN2 that were significantly different (*P* < 0.05) relative to the PC and were relevant to the study. The y-axis lists the metabolic pathways, while the x-axis represents pairwise comparisons among treatment groups. Triangle size is proportional to the absolute log₂ fold change, indicating the magnitude of the change in pathway abundance. The color of each triangle denotes the primary bacterial species contributing to the observed differential abundance. The direction of each triangle corresponds to the regulation of the pathway, where upward-pointing triangles indicate upregulation and downward-pointing triangles indicate downregulation. All comparisons use the PC as the reference, except for the first comparison (PC vs. NC), where the NC serves as the reference, and the direction of regulation reflects changes in the PC. Treatment groups include: PC, Positive Control (challenged); OH, Oat Hulls; SH, Soy Hulls; OH-WM, Oat Hulls with Wheat Middling; OH-SBP, Oat Hulls with Sugar Beet Pulp; SH-WM, Soy Hulls with Wheat Middling; and SH-SBP, Soy Hulls with Sugar Beet Pulp.

The enteric challenge also altered the functional potential of the cecal microbiome, shifting it from a primarily fermentative profile to one favoring bacterial growth and proliferation (Figure 6). Several fermentative and metabolic pathways were significantly (*P* < 0.001, unless indicated otherwise) reduced, including l-arginine degradation XIII (log₂FC = −1.49), primarily driven by *Escherichia coli*, methanogenesis from acetate (log₂FC = −2.30), and glycerol degradation to 1,3-propanediol (log₂FC = −1.58). In contrast, pathways associated with bacterial growth, membrane synthesis, and virulence were enriched. These included super pathway of menaquinol biosynthesis (log₂FC = 0.81), driven by *E. coli*, the super pathway of phospholipid biosynthesis (log₂FC = 0.74), primarily attributed to *E. coli*, and aerobactin biosynthesis (log₂FC = 1.50, *P* = 0.02), contributed by *Klebsiella oxytoca*.

Fiber supplementation modulated the functional profile of challenged birds, generally shifting it back toward a fermentative and biosynthetic state. The OH treatment increased the pentose phosphate pathway (non-oxidative branch) (log₂FC = 0.52, *P* = 0.011) and dTDP-N-acetylthomosamine biosynthesis (log₂FC = 1.60, *P* = 0.002), primarily driven by *Bacteroides cellulosilyticus* and *Bacteroides fragilis*. This also enhanced seleno-amino acid biosynthesis (log₂FC = 0.55, *P* = 0.022) and l-threonine biosynthesis (log₂FC = 0.61, *P* = 0.026), with *B. fragilis* as a key contributor. The combination of OH and WM supplementation enriched the super pathway of l-cysteine biosynthesis (log₂FC = 1.51, *P* = 0.012), attributed to *Bacillus sporothermodurans*. The combination of OH and SBP supplementation enriched the Bifidobacterium shunt pathway (log₂FC = 1.61, *P* = 0.008), driven by *Lactobacillus fermentum*, and heterolactic fermentation (log₂FC = 1.07, *P* = 0.016), attributed to *E. coli*. SH supplementation increased (S)-propane-1,2-diol degradation (log₂FC = 1.48, *P* < 0.001), driven by *E. coli*, and l-tyrosine degradation I (log₂FC = 4.55, *P* = 0.033). In challenged birds, SH combined with WM significantly increased sucrose degradation IV (log₂FC = 1.70, *P* < 0.0001), driven by *E. coli* and *Lactobacillus crispatus*; stachyose degradation (log₂FC = 1.02, *P* = 0.003), driven by *Lactobacillus salivarius*; and pyruvate fermentation to acetate and lactate (log₂FC = 0.81, *P* = 0.038), also attributed to *L. salivarius*. The combination of SH with SBP significantly enriched the super pathway of l-cysteine biosynthesis (log₂FC = 1.98, *P* < 0.001), attributed to *B. sporothermodurans*. A common finding across SH and SH-WM was an increase in tetrapyrrole biosynthesis I, a pathway important for vitamin B12 (log₂FC = 0.73, *P* = 0.002), mainly driven by *E. coli* and *Flavonifractor* species. In addition, the l-arginine degradation XIII pathway was increased in OH-SBP (log₂FC = 0.74, *P* = 0.033) SH-SBP (log₂FC = 0.72, *P* = 0.037) and SH-WM (log₂FC = 1.10, *P* = 0.001), with *E. coli* as the main contributor.

### Cecal mucosal transcriptome

**Fig. 7:**
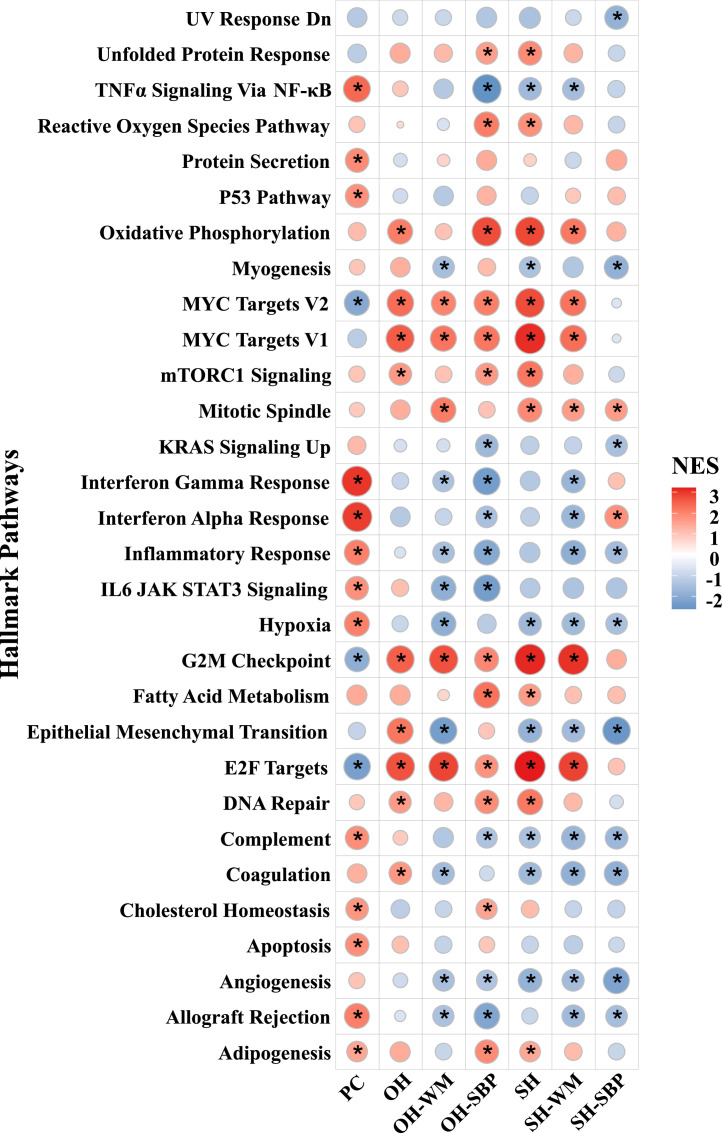
Gene Set Enrichment Analysis (GSEA) of Hallmark pathways in host cecal tissue. This bubble heatmap displays the top 30 enriched pathways from GSEA based on the absolute NES score. The y-axis lists the Hallmark gene sets, and the x-axis represents the comparisons of the treatment groups. The color of each bubble corresponds to the direction of enrichment, where red indicates pathways upregulated and blue indicates pathways downregulated relative to the PC, except for the first comparison (PC vs. NC), in which the NC serves as the reference and the direction of regulation reflects changes in the PC. The size of the bubble is proportional to the absolute value of the NES. Asterisks (*) denote a significant enrichment with a FDR q-value < 0.05. Treatment groups are as follows: PC, Positive Control (challenged); and challenged groups receiving diets containing OH, Oat Hulls; SH, Soy Hulls; OH-WM, Oat Hulls with Wheat Middlings; OH-SBP, Oat Hulls with Sugar Beet Pulp; SH-WM, Soy Hulls with Wheat Middlings; and SH-SBP, Soy Hulls with Sugar Beet Pulp.

The RNA-Seq of cecal samples generated an average of approximately 24.3 million reads per sample, providing sufficient sequencing depth for reliable differential gene expression analysis (Figure S4). The enteric challenge induced significant changes in the cecal transcriptome. There were 126 DEGs (*P_adj_* < 0.05) in the cecum between the challenged and the unchallenged groups (Table 2). Of these, 104 DEGs were upregulated and 22 were downregulated in the PC group. Gene Set Enrichment Analysis with Hallmark gene sets (Figure 7) revealed a significant enrichment (FDR < 0.01) of the interferon-gamma response (NES = +2.96), interferon-alpha response (NES = +2.93), and tumor necrosis factor alpha (**TNFα**) signaling via nuclear factor kappa B (**NF-κB**) (NES = +2.36) pathways in response to the enteric challenge. Pathways associated with cellular proliferation were significantly suppressed in the cecum, including transcription factor (**E2F**) targets (NES = −2.31), proto-oncogenes (**MYC**) targets V2 (NES = −2.06), and the G-phase to M-phase (**G2M**) checkpoint (NES = −1.94).

**Table 2:** Differentially expressed genes (DEGs) in the cecal mucosa of broilers provided diets with different dietary fiber sources on d 21.

Comparison^1^	DEGs^2^	Upregulated^3^	Downregulated^4^
PC vs NC	126	104	22
OH vs PC	0	0	0
OH-WM vs PC	0	0	0
OH-SBP vs PC	0	0	0
SH vs PC	0	0	0
SH-WM vs PC	0	0	0
SH-SBP vs PC	0	0	0

1 All treatments except NC were challenged with *Eimeria* spp. on d 14 and 10⁸ CFU of C. perfringens on d 18, and samples were collected at d 21.

2 Differentially expressed genes (DEGs) have been filtered based on P_adj_ < 0.05 and LogFC > ±1.

3 Upregulated genes have a log fold change of greater than +1 (LogFC > +1).

4 Downregulated genes have a log fold change of less than −1 (LogFC < −1).

Supplementation with dietary fiber modulated (FDR < 0.01, unless indicated otherwise) the cecal transcriptomic response to the enteric challenge. The inclusion of OH in the challenged group upregulated the pathways related to cellular proliferation, including E2F targets (NES = +2.70), G2M checkpoint (NES = +2.55), and MYC targets V1 (NES = +2.54). Additionally, pathways for oxidative phosphorylation (NES = +2.09) and the epithelial-mesenchymal transition (NES = +2.16) were also significantly enriched. No Hallmark gene sets were suppressed in the OH group compared to the PC. The combination of OH and WM downregulated several pathways, including the epithelial-mesenchymal transition (NES = −2.35), IL-6 (Interleukin-6)/JAK (Janus Kinase)-STAT3 (Signal Transducer and Activator of Transcription 3) signaling (NES = −1.88; FDR = 0.002), inflammatory response (NES = −1.54; FDR = 0.038), and interferon-gamma response (NES = −1.47; FDR = 0.046). Concurrently, there was upregulation of pathways such as E2F targets (NES = +2.81), G2M checkpoint (NES = +2.70), and MYC targets V1 (NES = +2.20). The addition of OH with SBP showed a suppression of pathways including TNFα signaling via NF-κB (NES = −2.61), interferon-gamma response (NES = −2.41), and IL-6/JAK-STAT3 signaling (NES = −2.36). Simultaneously, there was an enrichment of pathways, including oxidative phosphorylation (NES = +2.74), fatty acid metabolism (NES = +2.22), MYC targets V1 (NES = +2.15), and the G2M checkpoint (NES = +1.90; FDR < 0.01).

Supplementation with SH alone in the challenged group enriched the pathways, including E2F targets (NES = +3.17), G2M checkpoint (NES = +3.09), MYC targets V1 (NES = +3.08), and oxidative phosphorylation (NES = +2.78). This group also had a downregulation of pathways, including the epithelial-mesenchymal transition (NES = −1.84; FDR = 0.008) and TNFα signaling via NF-κB (NES = −1.64; FDR = 0.016). The combination of SH and WM suppressed multiple inflammatory pathways, including the inflammatory response (NES = −1.90; FDR = 0.004), interferon-gamma response (NES = −1.76; FDR = 0.005), and TNFα signaling via NF-κB (NES = −1.57; FDR = 0.016). Concurrently, there was upregulation of pathways such as G2M checkpoint (NES = +3.06), E2F targets (NES = +2.86), MYC targets V1 (NES = +2.30), and oxidative phosphorylation (NES = +2.14; FDR < 0.01). Finally, the combination of SH with SBP significantly suppressed pathways such as epithelial-mesenchymal transition (NES = −2.52) and angiogenesis (NES = −2.24), while upregulating the interferon-alpha response (NES = +1.76; FDR = 0.01) and the mitotic spindle pathway (NES = +1.60; FDR = 0.025).

### Correlation between bacteriome and host response

**Fig. 8:**
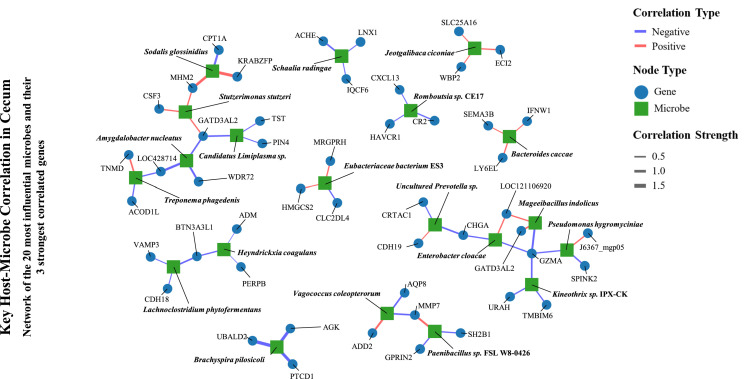
Network of the 20 most influential bacterial hubs and their primary host gene associations. This network visualizes the key interactions in the broiler cecal bacteriome. The 20 bacteria with the strongest individual association coefficients in the dataset were first identified as “hubs” (green squares). For each of these bacterial hubs, lines (edges) connect to the three host genes (blue circles) with which they have the strongest positive (red edges) or negative (blue edges) association. The width of each edge is proportional to the absolute magnitude of the LASSO coefficient, indicating the strength of the interaction.

Procrustes analysis revealed a trend toward a global association between the cecal bacteriome and host gene expression (correlation = 0.377, *P* = 0.073). To further explore this relationship, sCCA identified a stronger canonical correlation (r = 0.585) between a specific set of bacterial taxa and host genes. To identify specific one-to-one associations, a gene-by-gene Lasso regression was performed, and the resulting significant pairings were visualized as a host-bacterial interaction network (Fig. 8). The network displayed a distributed structure, with most bacteria linked to unique host genes. A predominance of negative correlations was observed, suggesting widespread antagonistic or competitive host-bacterial dynamics in the cecum.

Within this network, several host genes functioned as key hubs. For example, GATD3AL2, involved in protein deglycation, was positively correlated with bacterium *Stutzerimonas stutzeri* but negatively associated with *Amygdalobacter nucleatus* and *Candidatus Limiplasma* sp*.* Similarly, the immune effector gene GZMA exhibited multiple negative correlations, including with *Pseudomonas hygromyciniae* and *Enterobacter cloacae*, indicating its probable role in modulating antibacterial responses.

Fiber-degrading bacteria featured prominently, showing distinct associations with host functional pathways. *Bacteroides caecae*, a primary fiber degrader, correlated positively with immune regulators (IFNW1, LY6EL) and the neural guidance molecule SEMA3B. *Lachnoclostridium phytofermentans*, a plant polysaccharide degrader, showed negative associations with cell adhesion (CDH18) and vesicle trafficking (VAMP3) genes. *Prevotella sp.* displayed opposing correlations positive with CDH19 but negative with the enteroendocrine marker CHGA. *Eubacteriaceae bacterium* ES3 was positively linked with HMGCS2, a key enzyme in ketogenesis, potentially linking bacterial fermentation with host energy metabolism.

## Discussion

The fiber ingredients used in the present study are not typically included directly in commercial poultry diets at the evaluated inclusion levels. However, they were selected as representative fiber sources because of their distinct soluble and insoluble fiber composition profiles, particularly differences in fermentability and physicochemical properties within the gastrointestinal tract. These ingredients also represent commonly available feed commodities or byproducts that can be readily sourced and incorporated into practical feed formulations when targeting specific dietary fiber characteristics.

Using these representative fiber sources, the present study evaluated how dietary fiber composition influenced the cecal microbial response during enteric challenge. The co-infection with *Eimeria* spp. and *C. perfringens* in the challenged positive control group significantly impacted the cecal bacteriome, reducing bacterial diversity and evenness, while altering its community composition and function. This disruption provided a mechanistic basis for understanding how subclinical enteric infection compromised gut health and impaired growth performance in broilers. The challenge-induced microbial shift was characterized by significant depletion of butyrate-producing bacteria, including *Faecalibacterium prausnitzii, Anaerobutyricum hallii*, and *Coprococcus* sp., alongside enrichment of *Bacteroidales* members such as *Bacteroides fragilis* and *Phocaeicola vulgatus*. A microbial shift is a well-documented phenomenon in avian necrotic enteritis models, where enteric infections disrupt the balance of gut microbiota, creating an environment that favors the proliferation of opportunistic species (Wu et al., 2014; Stanley et al., 2014). The depletion of butyrate-producing bacteria can particularly be detrimental to intestinal homeostasis, as butyrate serves as the primary energy source for colonocytes and fulfills a major portion of the colonic epithelial energy demand (Guilloteau et al., 2010).

The challenge also reduced microbial fermentative pathways while enriching biosynthetic pathways for menaquinol, phospholipids, and aerobactin, primarily driven by Enterobacteriaceae. This metabolic shift from fermentation toward microbial biosynthesis and virulence-associated pathways mirrors observations in inflammatory bowel conditions where oxygen and nitrate availability favor facultative anaerobes capable of respiration (Winter et al., 2013; Rivera-Chávez et al., 2016). The enrichment of aerobactin biosynthesis is particularly interesting, as this siderophore enhances bacterial iron acquisition and has been associated with virulence in enteric pathogens (Ellermann and Arthur, 2017).

Transcriptomic analysis revealed that this microbial disruption coincided with host responses. The challenge induced significant upregulation of interferon-gamma, interferon-alpha, and TNFα signaling via NF-κB pathways, representing the classical Th1-type defense against intracellular protozoa such as *Eimeria*. This immune response is well-documented, as IFN-γ is recognized as a signature cytokine mediating Th1 immune responses in avian coccidial infections (Lillehoj and Trout, 1996; Kim et al., 2019a; Gaghan et al., 2022). Concurrently, pathways associated with cellular proliferation were significantly suppressed, including E2F targets, MYC targets, and the G2M checkpoint. This dual pattern of increased inflammation and suppressed proliferation aligns with observations that inflammatory signaling can redirect cellular resources away from growth processes toward immune defense (Klasing, 2007). The metabolic cost of this immune activation likely diverted energy away from anabolic functions, providing a molecular link between the enteric challenge and the performance impairments.

Dietary fiber supplementation altered the pathological shifts in challenged groups, though the mechanisms varied by fiber source and combination. The supplementation of OH alone in the challenged group increased the abundance of *Brevibacterium linens* while decreasing several species, including *Phocaeicola vulgatus, Prevotella melaninogenica*, and *Prevotella nigrescens*. The reduction *in P. vulgatus* is important, as some strains of this species have been associated with inflammatory conditions in mammalian models and can produce immunostimulatory lipopolysaccharides (Di Lorenzo et al., 2020). Functionally, OH supplementation increased the pentose phosphate and dTDP-N-acetylthomosamine biosynthesis pathways, mainly driven by *Bacteroides fragilis* and *E. coli*. The pentose phosphate pathway supports NADPH production for antioxidant defense during infection-induced oxidative stress (Kwon et al., 2019; Shekhova, 2020). It also enhanced seleno-amino acid and l-threonine biosynthesis, with *B. fragilis* contributing to selenoprotein formation that protects against oxidative damage and supports immune function (Habte-Tsion et al., 2016; Zhang et al., 2020). The host transcriptional response to OH supplementation also showed activation of proliferative and metabolic pathways, including E2F targets, G2M checkpoint, MYC targets, and oxidative phosphorylation, indicating enhanced cell renewal and energy production for tissue repair. However, OH did not suppress inflammatory signaling compared to the challenged control, suggesting that while it supported epithelial recovery, additional modulation of inflammation may be required to prevent immunopathology.

The combination of OH and WM restored butyrate-producing bacteria, markedly increasing *F. prausnitzii, Candidatus Faecalibacterium intestinigallinarum*, and *Eubacterium limosum* while reducing *C. perfringens* abundance. This simultaneous enrichment of potentially beneficial microbes and suppression of pathogen indicates a favorable shift in the gut microbiome. *F. prausnitzii* is a major butyrate producer whose abundance inversely correlates with intestinal inflammation across species (Miquel et al., 2013). Functionally, OH-WM supplementation enriched l-cysteine biosynthesis, supporting glutathione production, the main intracellular antioxidant, which helps protect cells from infection-induced oxidative stress (Jiao et al., 2023). It also restored l-arginine degradation pathways, promoting polyamine production that supports epithelial proliferation, differentiation, and barrier integrity (Nakamura et al., 2021). OH-WM supplementation suppressed inflammatory pathways, including IL-6/JAK-STAT3 signaling, interferon-gamma response, and epithelial-mesenchymal transition, reducing inflammation and tissue remodeling (Dann et al., 2008; Li et al., 2016). At the same time, it upregulated E2F targets and G2M checkpoint pathways, restoring epithelial cell proliferation essential for barrier maintenance and intestinal recovery (Chong et al., 2009; Williams et al., 2015; Walston et al., 2021).

In contrast, the OH-SBP combination promoted several lactic acid producing taxa, along with *Bifidobacterium animalis*. The pectin-rich soluble fibers present in sugar beet pulp are likely a substrate for these bacteria. This observation aligns with previous reports showing selective enrichment of *Bifidobacterium* in response to pectic substrates under both *in vivo* and *in vitro* conditions, and a similar trend was observed here under enteric challenge in broilers. (Vigsnæs et al., 2011; Gómez et al., 2016; Yu et al., 2019; Lysko et al., 2022). Functionally, OH-SBP enriched the Bifidobacterium shunt pathway and heterolactic fermentation. The bifid shunt is a specialized glycolytic pathway unique to bifidobacteria that produces acetate and lactate (Pokusaeva et al., 2011), while heterolactic fermentation by *lactobacilli* generates lactate, acetate, and other organic acids (Salminen and Wright, 2004). In the host tissue, OH-SBP supplementation markedly suppressed key inflammatory pathways, including TNFα via NF-κB, interferon-gamma, and IL-6/JAK-STAT3 signaling, reducing pro-inflammatory cytokine production and potentially supporting tight junction proteins to improve barrier integrity (Ma et al., 2004). Further, this treatment enhanced oxidative phosphorylation and fatty acid metabolism, potentially increasing mitochondrial ATP generation and supporting the energetic demands of epithelial cells during stress, thereby promoting tissue repair and maintaining gut homeostasis (Mitchell, 1961; Byndloss et al., 2017).

Supplementation of SH alone significantly increased *Clostridia bacterium* UC5.1-1D4, *Ruminococcus* sp. SR1/5, and *Agathobaculum* sp., all of which are fiber degrading (Christopherson et al., 2014; Ahn et al., 2016; Limsrivilai et al., 2025), and are recognized for their capabilities of producing short-chain fatty acids. Further, there was a decrease in the abundance of *B. thetaiotaomicron, Phocaeicola vulgatus, Butyricimonas faecihominis*, and *Enterococcus avium*. Functionally, SH supplementation increased propane-1,2-diol degradation and l-tyrosine degradation pathways, primarily driven by *E. coli*. Propanediol degradation can yield propionate, a short-chain fatty acid with anti-inflammatory properties (Ciarlo et al., 2016). The host transcriptional response to SH showed enrichment of E2F targets, G2M checkpoint, MYC targets, and oxidative phosphorylation pathways, alongside downregulation of epithelial-mesenchymal transition and TNFα signaling via NF-κB. This pattern indicates enhanced proliferative capacity with moderate anti-inflammatory effects.

Soy hulls, particularly when combined with wheat middling, promoted a microbial profile similar to that observed in the OH-SBP group, characterized by a predominance of lactic acid bacteria. Soy hulls contain non-starch polysaccharides (**NSP**), including cellulose and pectin, which serve as fermentable substrates for lactic acid bacteria (Yang et al., 2020b), consistent with similar enrichment reported in swine (Qiu et al., 2024). However, as NSP have also been identified as a predisposing factor for NE, the observed bloom of lactobacilli may, in part, reflect an opportunistic response under challenge conditions (Liu et al., 2010; Sun et al., 2015; Gharib-Naseri et al., 2019). Functionally, SH-WM enhanced pyruvate fermentation to acetate and lactate, alongside pathways for degrading sucrose and stachyose. Stachyose is a tetra saccharide abundant in soy products that can be fermented by lactobacilli but not by the host or many pathogenic bacteria. Fermentation of stachyose produces organic acids that lower luminal pH and inhibit pathogen growth (Zhao et al., 2021). The host transcriptional response to SH-WM demonstrated broad suppression of inflammatory pathways, including the inflammatory response, interferon-gamma response, and TNFα signaling, alongside upregulation of G2M checkpoint, E2F targets, and MYC targets. Across SH and SH-WM treatments, a consistent enrichment of tetrapyrrole biosynthesis pathways was observed. Tetrapyrroles serve as precursors for vitamin B12, which is synthesized exclusively by microorganisms and functions as a cofactor in one-carbon metabolism (Fang et al., 2017). In the gut, competition for B12 can influence microbial community composition, with some pathogens requiring this vitamin for growth while beneficial bacteria can synthesize it.

Interestingly, the cecal mycobiome remained stable despite substantial bacterial changes, with *Agaricus bisporus* maintaining dominance across treatments. This stability contrasts with bacterial volatility and suggests that fungal communities may either not noticeably respond to environmental changes or slower. Similar patterns have been observed in mammalian systems where bacterial shifts do not necessarily correlate with fungal community disruption (Mar et al., 2016). However, specific fiber treatments modulated fungal population slightly, with OH-SBP increasing *Malassezia restricta* and *Aspergillus chevalieri. Malassezia* species can interact with host immune cells through C-type lectin receptors and may influence immune development (Ishikawa et al., 2013), though the functional significance of these shifts in the avian gut requires further study.

The cecal virome showed slight treatment-specific changes in bacteriophage populations, with OH increasing an *Escherichia* phage and combination treatment enriching other phage taxa. Bacteriophages regulate bacterial population dynamics through lytic and lysogenic life cycles and can mediate horizontal gene transfer (Mirzaei and Maurice, 2017). Studies have demonstrated that phages can suppress pathogenic bacterial blooms and contribute to microbiome stability (Hsu et al., 2019; Kosznik-Kwaśnicka et al., 2022). The fiber-associated phage changes may reflect altered bacterial host populations, as phage abundance often correlates with host bacterial density, or potentially direct effects on phage-bacteria interactions.

Integration of bacteriome and transcriptome data through canonical correlation analysis revealed coordinated relationships between specific bacterial taxa and host genes. *Stutzerimonas stutzeri* correlated positively with CSF3, which encodes granulocyte colony-stimulating factor involved in neutrophil development (Lieschke et al., 1994). Several taxa showed negative correlations with GZMA, which encodes granzyme A, a serine protease involved in cytotoxic lymphocyte function (Duquette et al., 2023), suggesting that certain microbial community structures associate with reduced cytotoxic immune activity. *Eubacteriaceae bacterium* ES3 correlated positively with HMGCS2, which encodes an enzyme in ketogenesis (Cotter et al., 2014), and ketone body production in colonocytes has been linked to butyrate oxidation and energy metabolism (Donohoe et al., 2011). These correlations identify potential host-microbe interaction networks, though establishing causality would require mechanistic studies with defined bacterial communities or isolated bacterial products, as such associations could reflect bacteria influencing host gene expression, host genes creating environments favoring certain bacteria, or both responding to common dietary factors.

Overall, these findings reveal how different fiber sources affect the gut environment through distinct but complementary mechanisms under a challenge condition. All fiber combinations had similar host responses, despite having distinct microbial compositions, notably reduced inflammatory signaling and enhanced epithelial proliferation. This provides a mechanistic framework for developing evidence-based nutritional strategies to enhance gut resilience in broiler production systems transitioning away from antibiotic use. While this study establishes links between fiber supplementation, microbial ecology, and host physiology, future work incorporating direct metabolomic measurements to validate predicted metabolite production, time-course sampling to establish temporal relationships between microbial and host changes, and functional validation of important transcriptomic results would further refine our understanding of the dynamic processes underlying fiber-mediated gut health modulation.

## CRediT authorship contribution statement

**R.W. Tabish:** Writing – original draft, Investigation, Formal analysis, Data curation. **Y. Lin:** Investigation. **S.J. Rochell:** Writing – review & editing, Supervision, Methodology, Conceptualization. **W.J. Pacheco:** Writing – review & editing, Conceptualization. **M.A. Bailey:** Investigation. **W.A. Dozier III:** Writing – review & editing, Project administration, Funding acquisition, Conceptualization. **K. Robinson:** Writing – review & editing, Conceptualization. **R. Hauck:** Writing – review & editing, Supervision, Methodology.

## Disclosures

The authors declare that they have no known competing financial interests or personal relationships that could have appeared to influence the work reported in this paper.

## Appendix Supplementary materials

Image, application 1

Image, application 2

Image, application 3

Image, application 4

## Footnotes

Supplementary material associated with this article can be found, in the online version, at doi:10.1016/j.psj.2026.107151.
