# Supplementary material for: Cecal metagenome and mucosal transcriptome of broilers after an enteric challenge and fed diets with different fiber types and concentrations1
Source: Poult Sci. 2026 May 22;105(9):107151. doi: 10.1016/j.psj.2026.107151 (PMC13266104; doi:10.1016/j.psj.2026.107151)
Supplement: Supplementary file 4 [file mmc4.docx]

**SUPPLEMENTARY FIGURES AND TABLES**

**Response of broilers subjected to an enteric challenge and fed diets with different fiber types and concentrations - part 2: cecal metagenome, and mucosal transcriptome^1^**

R. W. Tabish, Y. Lin, S. J. Rochell, W. J. Pacheco, M. A. Bailey, W. A. Dozier, III, F. J. Hoerr, K. Robinson, and R. Hauck

^^

| **Figure S1. Distribution of sequencing reads from broiler cecal content samples.** |
| --- |
| The histogram shows the total number of reads per sample (in millions) obtained from shotgun metagenomic sequencing. The dataset exhibited a right-skewed distribution, with a mean of 36.0 million reads and a median of 32.8 million reads per sample. The read counts ranged from 30.5 to 54.5 million. |


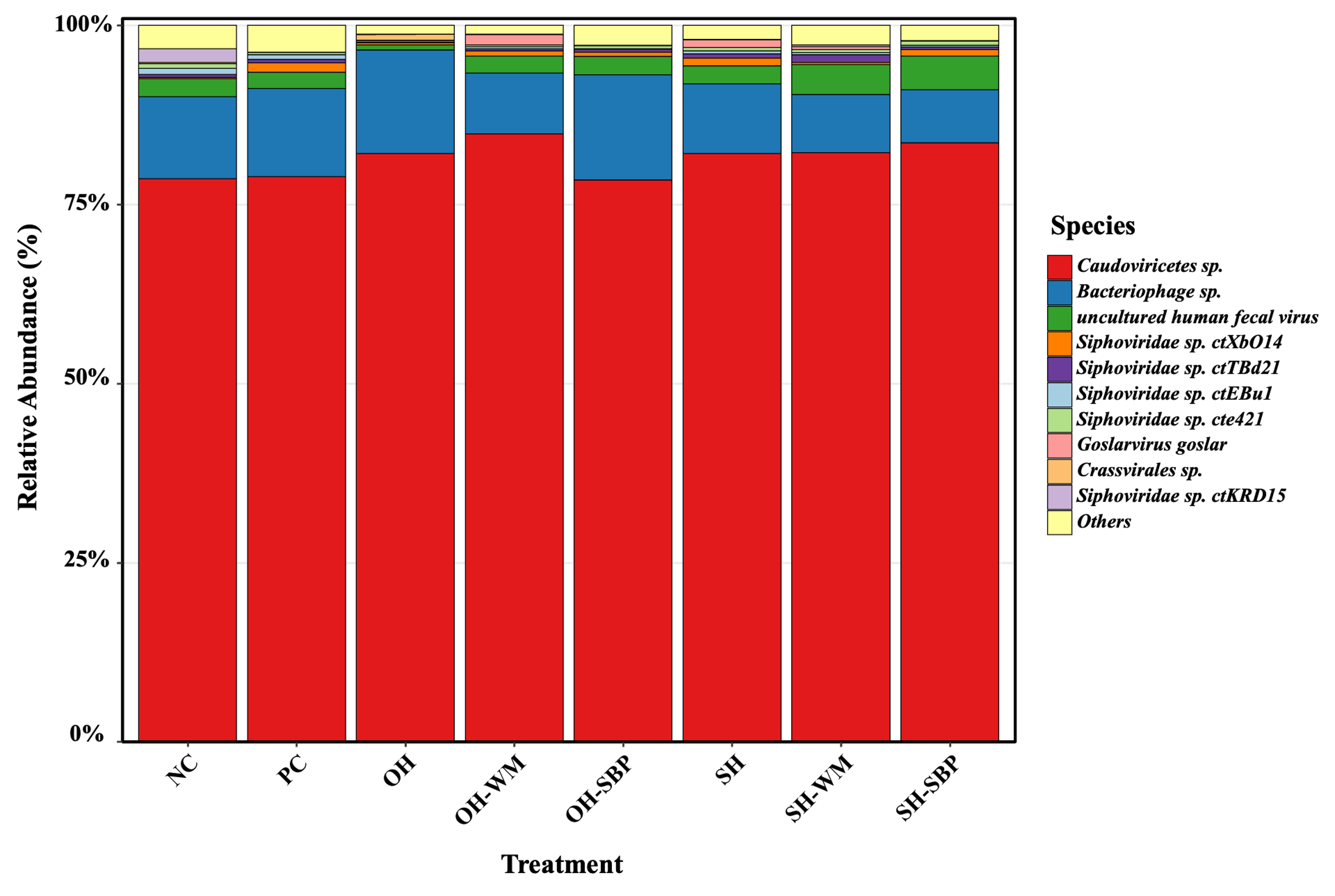


| **Figure S2. Relative abundance of the top 10 viral taxa in cecal samples across all treatment groups.** |
| --- |
| This stacked bar plot illustrates the mean relative abundance of the most prevalent viruses. Each bar represents a distinct treatment group, and the colored segments correspond to the relative abundance of the top 10 identified viral taxa, as indicated in the legend. The 'Others' category comprises all remaining, less abundant taxa not ranked in the top 10. Treatment groups are as follows: NC, Negative Control (unchallenged); PC, Positive Control (challenged); and challenged groups receiving diets containing OH, Oat Hulls; SH, Soy Hulls; OH-WM, Oat Hulls with Wheat Middling; OH-SBP, Oat Hulls with Sugar Beet Pulp; SH-WM, Soy Hulls with Wheat Middling; and SH-SBP, Soy Hulls with Sugar Beet Pulp. |


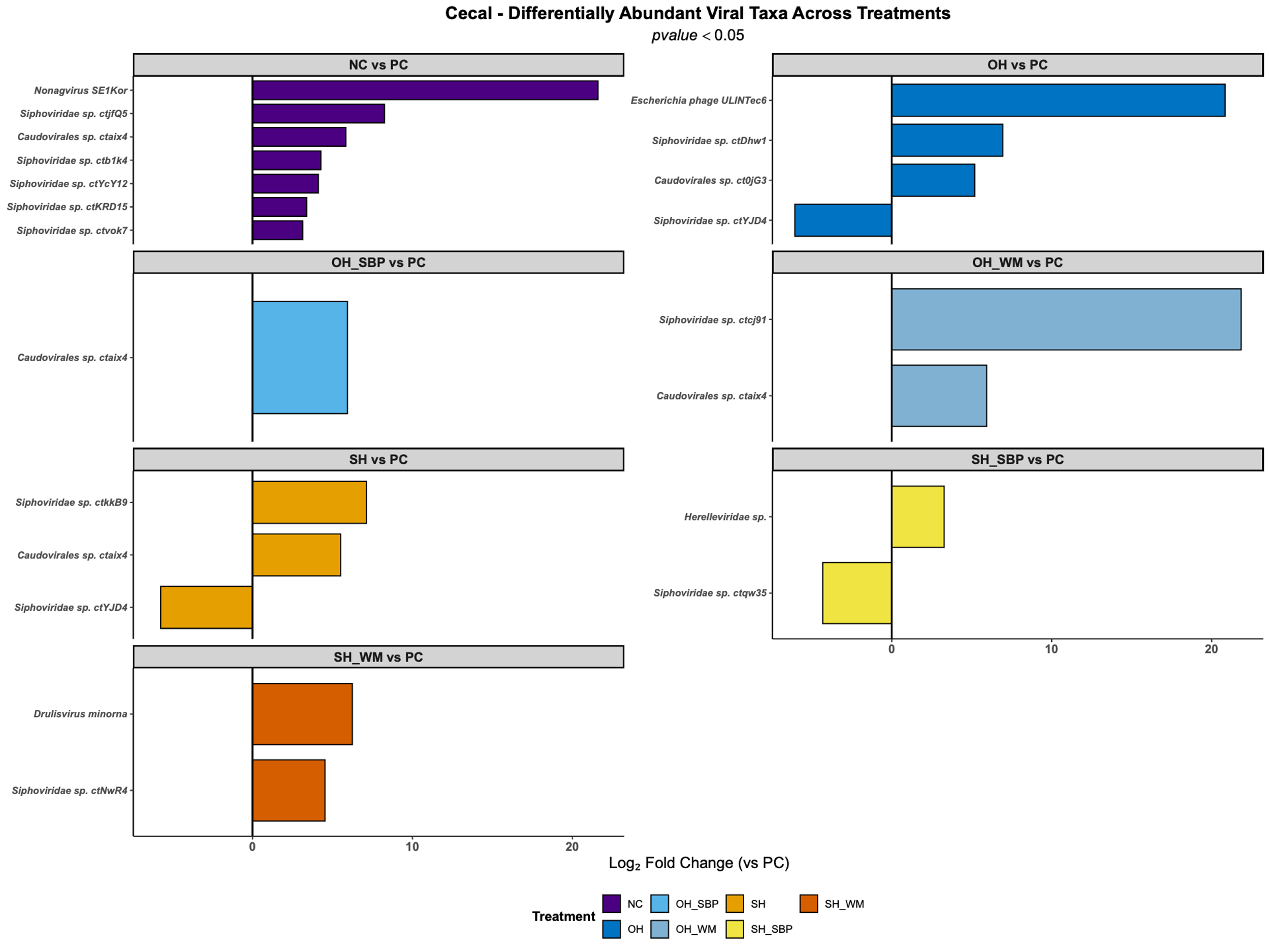


| **Figure S3. Differentially abundant viral taxa in the cecal contents of broilers across treatment groups.** |
| --- |
| The bar plots show the log₂ fold change of viral taxa on x-axis which were differentially abundant (P < 0.05) in each dietary treatment group compared to the challenged control (PC) group. Each panel represents a separate comparison. Treatment groups are as follows: NC, Negative Control (unchallenged); and challenged groups receiving diets containing OH, Oat Hulls; SH, Soy Hulls; OH-WM, Oat Hulls with Wheat Middling; OH-SBP, Oat Hulls with Sugar Beet Pulp; SH-WM, Soy Hulls with Wheat Middling; and SH-SBP, Soy Hulls with Sugar Beet Pulp. |

| **Figure S4. Distribution of raw sequencing read counts from broiler cecal mucosal samples.** |
| --- |
| The histogram illustrates the frequency distribution of read counts across all 72 RNA sequencing libraries. The x-axis represents the read count in millions (×10^6^), and the y-axis indicates the number of samples. The mean read count per sample was 24.3 ± 4.3 million (standard deviation), indicated by the red dashed line. The median was 24.4 million, and counts ranged from 12.5 to 38.9 million reads. |
